# Detection of remnants in clipped unruptured intracranial aneurysms by intraoperative CT-angiography and postoperative DSA: clinical relevance and follow-up

**DOI:** 10.1007/s00701-025-06518-3

**Published:** 2025-04-17

**Authors:** Jun Thorsteinsdottir, Julian Schwarting, Robert Forbrig, Sebastian Siller, Joerg-Christian Tonn, Thomas Liebig, Christian Schichor

**Affiliations:** 1 Department of Neurosurgery, LMU University Hospital, LMU Munich, Marchioninistr. 15, 81377 Munich, Germany; 2 Institute of Diagnostic and Interventional Neuroradiology, Klinikum Rechts Der Isar, Technical University Munich, Ismaninger Str. 22, 81675 Munich, Germany; 3 Institute for Stroke and Dementia Research (ISD), LMU University Hospital, LMU Munich, Feodor-Lynen-Straße 17, 81377 Munich, Germany; 4 Institute of Neuroradiology, LMU University Hospital, LMU Munich, Marchioninistr. 15, 81377 Munich, Germany; 5 Department of Neurosurgery, University of Regensburg, Franz-Josef-Strauß-Allee 11, 93053 Regensburg, Germany

**Keywords:** Cerebral aneurysm, Clipping, Intraoperative computer tomography, Postoperative angiography

## Abstract

**Background:**

Aneurysm clipping is routinely performed with high efficacy and low complication rates in specialized neurovascular centers. Postoperative aneurysm remnants bear the risk of growth/rupture. Study aim was to analyze remnants in postoperative angiography (pDSA) and follow-up (FU) and to evaluate whether use of intraoperative CT-angiography (iCTA) can intraoperatively detect remnants and enable therapeutic consequences.

**Methods:**

All patients undergoing elective aneurysm clipping at our center between 11/2012 and 12/2019 were included for FU in 01/2024. All patients received Indocyanin-green-videoangiography (ICGVA) and postoperative angiography (pDSA). After iCTA implementation in 10/2016, the majority of patients received additionally iCTA. Baseline characteristics, treatment-related morbidity/outcome, resulting operative conclusions in distinct cohorts with/without iCTA, and management of remnants according to Sindou classification were analyzed.

**Results:**

270 patients (367 enrolled/97 excluded) were clipped using iCTA in 74 patients. In 12/270 patients (4.5%) clip repositioning was performed due to ICGVA results, but iCTA further detected large remnants intraoperatively in 3/74 patients (4.1%) correctly resulting in re-clipping in two patients and recommendation for endovascular therapy in one patient. The specificity, sensitivity, and accuracy for detection of Sindou grade (SG) III-IV remnants by iCTA were 100%, 75%, and 98.6%, respectively. Overall, pDSA detected SG I-II remnants in 32/270 (11.9%) and SG III-V remnants in 8/270 (3.0%) patients with 3/270 requiring retreatment (*n* = 1 resurgery, *n* = 2 endovascular therapy). Frequency of SG I-V and III-V remnants were slightly lower in iCTA than non-iCTA group (10.8 vs. 16.3%, *p* < 0.173 and 1.4 vs. 3.6%, *p* < 0.306). All SG I-II and five SG III-V remnants did not reveal growth/rupture after a mean FU of 29 months.

**Conclusions:**

Aneurysm remnants after clipping are rare and predominantly small (SGI-II)—not harbouring a risk of growth/rupture during short-term FU. Intraoperative CTA can detect large aneurysm remnants (SG III-IV) and may prompt adjustment of surgical strategy in individual cases.

**Supplementary Information:**

The online version contains supplementary material available at 10.1007/s00701-025-06518-3.

## Introduction

Aneurysm clipping is routinely performed with high efficacy and low complication rates in specialized neurovascular centers [1, 2]. Up to now, microsurgical clip ligation leads to high occlusion rates. Residual aneurysms have been reported to be present between 3.8% and 18.2% of clipping procedures highly dependent on the sensitivity of the imaging modality for postoperative visualization [22, 33]. While the risk of recurrence of a presumably completely occluded intracranial aneurysm is reported to be low (0%–2.4%), approximately 10% of aneurysm remnants after clipping can exhibit future growth [4, 5, 46]. Especially, the presence of an aneurysm remnant on postoperative angiography may have far-reaching clinical consequences with a risk of rebleeding of 1.9% per year [11]. Therefore, intra- or postoperative detection of significant remnants is crucial. In the last decades, intraoperative diagnostic tools, particularly Indocyanin-green videoangiography (ICGVA) and intraoperative angiography were introduced to minimize the number of aneurysm remnants and demonstrate parent vessel patency [34, 35, 49]. However, routine use of intraoperative angiography can be technically and logistically challenging for many institutions [9].

For verification of postoperative complete aneurysmal obliteration digital subtraction angiography (DSA) is most accurate and remains the gold standard up to now [19, 43]. However, in rare cases DSA can cause transient or permanent neurological complications as a direct consequence of the procedure [10]. As an alternative examination, computed tomographic angiography (CTA) is less invasive and increasingly used to confirm obliteration of aneurysms post clipping [20, 47]. Previously published meta-analyses showed that CTA had favorable specificity, but less favorable sensitivity [43, 47]. For example, Uricchio et al. included 13 studies with a total of 613 aneurysms with the pooled analysis confirming CTA to have a sensitivity of 69% and a specificity of 99% for identifying a residual aneurysm. [47] The authors concluded that CTA may be applicable to rule in, but not to rule out an aneurysm remnant after clipping. However, this study did not address the remnant size in detail and up to now, results of clipping procedures using intraoperative CTA to detect aneurysm remnants are missing [12, 13].

Recently, we demonstrated that intraoperative computed tomography (iCT) including angiography (iCTA) and perfusion (iCTP) is feasible and provides valuable information without disturbing the surgical workflow [37, 38]. Modern iCT systems provide similar image resolution as conventional CT scanners of radiology departments, including the possibility of performing iCTA/iCTP and software for artifact reduction [16]. In vascular surgery, iCTA allows to define the parent vessel anatomy post-clipping while iCTP enables visualization of critical distant perfusion impairment in case of clip stenosis [39]. Recently, we showed in a large cohort that there was a stepwise improvement in the rate of complete aneurysm occlusion, postoperative ischemia and neurological outcome in the last 20 years [40]. However, in this former study not all patients received postoperative angiography and the remnant size and therapeutic consequences were not investigated in detail.

Therefore, aim of the present study was to evaluate whether the use of intraoperative CT-angiography can detect aneurysm remnants intraoperatively. Further, we analyzed in detail the remnant types according to the Sindou classification and observed the rate of growth and rupture in the follow-up (FU) time.

## Patients and methods

### Study design

Since 11/2012 all patients with microsurgical clipping of intracranial aneurysms received postoperative DSA as a standard. Therefore, all patients with unruptured aneurysm clipping between 11/2012 and 12/2019 at our center were retrospectively analyzed with last FU in 01/2024. Additionally, intraoperative CT, CTA and CTP was performed since 10/2016 according to the availability of the technical radiological assistant and specific requests of the vascular neurosurgeon. A subgroup of patients not receiving iCTA since 10/2016 was analyzed separately. Patients with ruptured intracranial aneurysms and contraindications/refusal of postoperative angiography were excluded. Throughout the whole observation period (2012–2019), indication for microsurgical clip occlusion was based on recommendations of our interdisciplinary neurovascular board in accordance with evidence-based guidelines for unruptured intracranial aneurysm (UIA) treatment of national and international specialist societies. Microsurgical clipping was performed by a specialized neurovascular team consisting of three staff neurosurgeons specialized in neuro-vascular microsurgery. Although the members of that group changed over time (but not in parallel to the above-mentioned subgroups/treatment eras), the qualification within the neurovascular team remained similar and part of that team remained the same. Clinical data, iCT including unenhanced brain CT, CTA and CTP, pre-/post-operative angiography and ICGVA as well as subsequent imaging data during in-hospital stay were retrospectively analyzed. Clipping results were analyzed based on iCTA and DSA, the latter being performed within 7 days after clipping (upon the availability of DSA performed by the neuroradiologists during the usual in-hospital stay of seven days). Clinical outcome was analyzed postoperatively and at last FU by Glasgow outcome scale (GOS) and modified Rankin scale (mRS). The study was approved by the institutional review board (No.19–560). Informed consent was obtained from all patients.

### Radiological Imaging

All patients underwent preoperative DSA, including 3D reconstruction. In cases of new postoperative deficits or prolonged wake-up periods, a CT scan with CT angiography and perfusion scan was performed immediately after surgery, otherwise, an unenhanced CT scan was performed on the first postoperative day in every case. Within 7 days of in-hospital stay, postoperative DSA with 3D reconstruction was performed and results were discussed at the interdisciplinary neurovascular board. Aneurysm remnants were classified according to the Sindou classification: Sindou Grade (SG) I: less than 50% of neck implantation, SG II: more than 50% of neck implantation, SG III: residual lobe from a multilobulated sac, SG IV: residual portion of the sac, less than 75% of aneurysmal size, SG V: residual portion of the sac more than 75% of aneurysmal size [41]. According to the aneurysm remnant size and configuration in postoperative DSA, indication for further treatment (surgical/endovascular) or FU by imaging (angiography, MRA, CT angiography) was determined by the interdisciplinary neurovascular board. In general, the following regimen for aneurysm remnants was recommended according to the interdisciplinary neurovascular board:

- SG I remnants or remnants < 1 mm: depending on configuration of the aneurysm remnant either no control, CTA (> 2 clips) or MRA (≤ 2 clips) after 5 years.
- SG II remnants or remnants < 2 mm: depends on configuration of the aneurysm either MRA/CTA (> 2 clips) or MRA (≤ 2 clips) after 2 years.
- SG III remnants or remnants ≤ 2 mm: depends on configuration of the aneurysm remnant either retreatment or CTA (> 2 clips), MRA (≤ 2 clips) or DSA (in complex clip reconstructions) after 2–3 years.
- SG IV or V remnants or remnants > 2 mm: retreatment by resurgery or endovascular therapy.

### Intraoperative CTA/CTP

Intraoperative CT including unenhanced brain CT, iCTA and iCTP was performed since 10/2016. Intraoperative imaging was analyzed on site immediately by an experienced neuroradiologist and pathological findings were reported immediately to the surgeon. In case of a perfusion deficit or aneurysm remnant, the clip was immediately repositioned. In case of local vasospasm detected by ICGVA and/or iCTA, nimodipine was administered locally and intravenously.

### Standardized iCT protocol/intraoperative workflow

ICT was performed using a Siemens SOMATOM Definition AS +, Siemens Healthineers (Siemens AG, Munich, Germany) as recently described [16, 29, 37–39]. Patients were positioned on carbon-made radiolucent surgical tables (TruSystem 7500, Trumpf Medical), the patient’s head was fixed in a radiolucent head clamp (Mayfield radiolucent skull clamp, A- 2002, Integra) and the table position was saved to avoid collision with the CT gantry [3]. Surgery was performed using continuous IONM if deemed necessary, ICGVA and microdoppler ultrasonography. Immediately after clip placement, the table was placed in the previously saved scanning position. Subsequently, CT and intravenous administration of 50 ml non-ionic iodinated contrast material (iomeprol; Imeron® 350 mg I/ml, Bracco Imaging) at a flow rate of 4.0 ml/s followed by a 50 ml saline flush were performed within 90–120 s. 3D postprocessing of iCTA/dynamic perfusion analysis of iCTP data set was achieved within 3 min by a technical assistant. After data acquisition/reconstruction (in total ~ 5 min), the neuroradiologist reviewed iCT and immediately reported any pathological findings to the neurosurgeon. Mismatch was defined as prolonged mean transit time (MTT) or Time to maximum (Tmax) with moderately reduced cerebral blood flow (CBF) and near-normal/increased cerebral blood volume (CBV).

According to intraoperative findings and/or CTA/CTP results, the clip was repositioned or conservative treatment regimen were applied (e.g. elevation of mean arterial pressure, application of nimodipine, anticonvulsive therapy, anticoagulation).

### Indocyanine green videoangiography (ICGVA)

All operations were performed using a microscope-integrated infra-red sensitive monochrome video camera (OPMI Pentero with INFRA-RED 800, Zeiss, Oberkochen, Germany). Fluorescent dye (indocyanine green, Verdy®; Diagnostic Green GmbH, Aschheim-Dornach, Germany) was administered intravenously (10 mg per dose, 0.2–0.5 mg/kg body weight) [34]. Images were continuously displayed to evaluate visualization quality and initial dye inflow. Additionally, local blood flow was assessed by microdoppler ultrasonography. ICGVA was applied in all patients without contraindications including diseases of liver or kidney. In case of an intraoperative pathology including aneurysm remnant or stenosis, the clip was repositioned and ICGVA was performed again to confirm a sufficient clipping result.

### Radiation exposure

Regarding radiation dose exposure analysis, we calculated the mean effective dose (ED) of both iCTA and postoperative angiography (routinely including biplane DSA covering the whole vessel territory, 3D rotational angiography and a zoomed-in DSA run targeting the parent vessel and aneurysm neck area thoroughly angled with respect to the aneurysm clip). In detail, by using the dedicated dose reports archived in the picture archiving and communication system (PACS), the mean dose length product (DLP) and dose area product (DAP), respectively, was multiplied with the conversion factor for head CT [16] and head DSA [44]. Hardware details and protocol settings of used iCT and angiography systems were reported elsewhere [16, 17].

### Statistical analysis

We performed power calculations to justify the sample size for detecting differences between subgroups using G*Power 3.1.9.7. Intra- and interrater agreement for Sindou grading classifications was analyzed using Kendall’s τ-b correlation coefficient.

Continuously scaled variables were analyzed with the Mann–Whitney U test, categorical variables with Chi-square or Fisher’s exact test. Factors associated with Sindou I-V and Sindou III-V remnants were identified using univariate analysis. Factors potentially affecting remnant detection were investigated in multivariate logistic regression analysis. A p-value ≤ 0.05 was considered significant. All calculations except power calculations were performed using SPSS software package (Version 25).

## Results

### Patient inclusion

In order to justify the sample size, we conducted a power calculation with a two-group comparison to detect a medium effect size (Cohens d = 0.5) with α = 0.05 and power = 0.95, and allocation ratio (iCTA/non-iCTA group) = 0.38. The standard power table suggested a total sample size group of *n* = 220 with *n* = 61 in the iCTA group and *n* = 159 in the non-iCTA group.

**Fig. 1:**
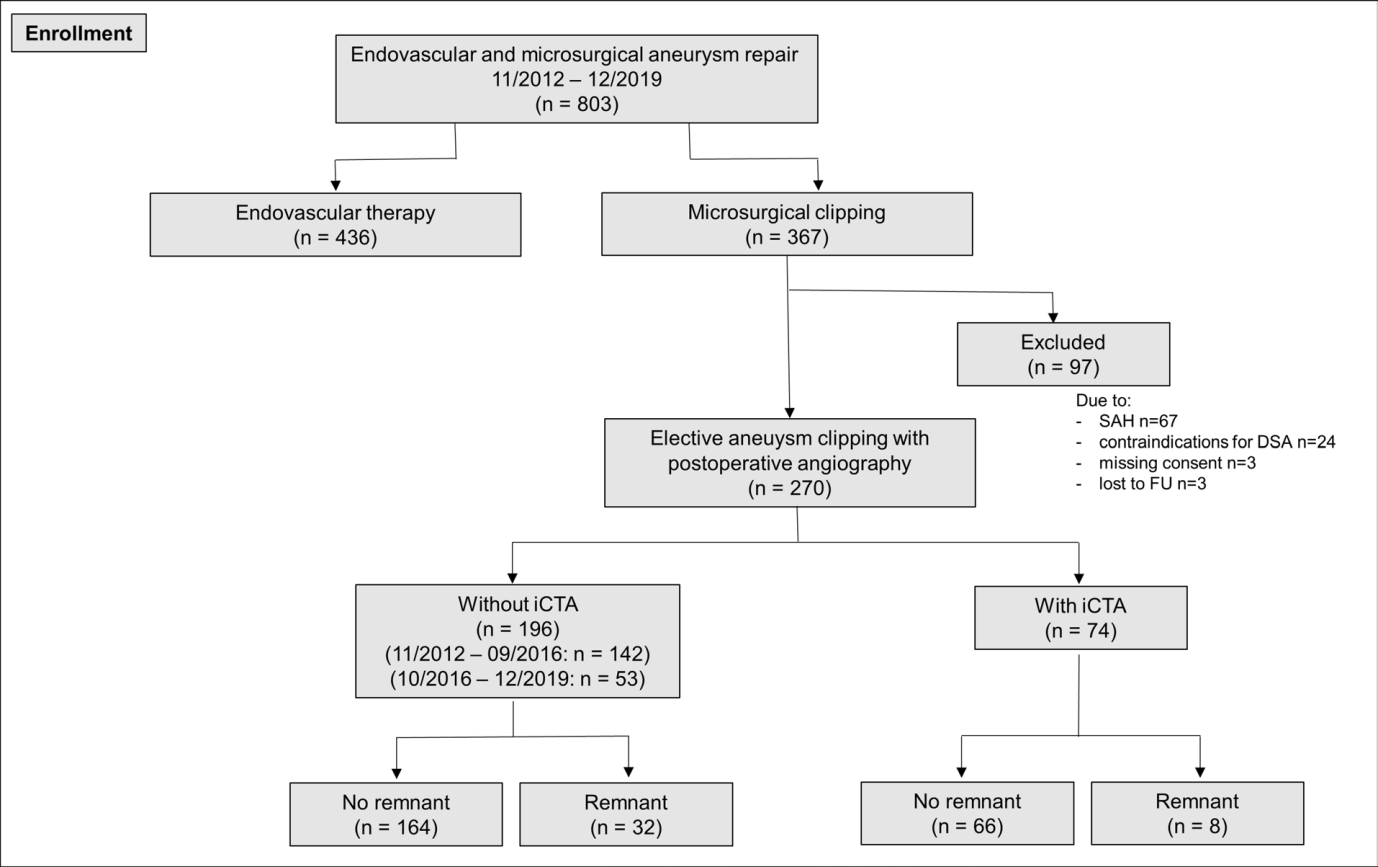
Flow-chart of patients’ inclusion for analysis. SAH: subarachnoid hemorrhage, iCT: intraoperative CT

Study reference point was the date of clipping. Last FU was in 01/2024. 367 patients have been operated on intracranial aneurysms between 11/2012 and 12/2019 at our center. Patients have been excluded because of prior subarachnoid hemorrhage (*n* = 67) and lack of postoperative DSA according to contraindications including allergy, renal dysfunction or other underlying diseases (*n* = 30), missing consent to DSA (*n* = 3) or lost to FU (*n* = 3). Therefore, 270 patients were included with 196 patients not receiving iCTA (non-iCTA group) and 74 patients receiving multimodal iCTA (since 10/2016) (Fig. 1). Among the 270 patients, a subgroup of 53 patients who were operated since 10/2016 without iCTA was analyzed separately.

### Patient characteristics

Median age was 57.0 years (range: 18—81 years) and 73% of the patients were female. Aneurysms were located at MCA (58.1%), ICA (11.5%), ACoA (18.9%), ACA (7.0%), and PCoA (3.0%), PICA (1.1%) and SUCA (0.4%). 31.5% of the patients exhibited multiple aneurysms. The location was on the right side in 34.4%, on the left side in 47.0% and in the middle in 18.5% in ACoA aneurysms and in 0.4% in an azygotic ACA aneurysm. Median aneurysm size was 6.7 mm. Temporary clipping was necessary in 40 patients (14.8%) with a median temporary clipping time of 8.0 min. Mean FU time was 28.9 months. Baseline characteristics did not differ significantly between the iCTA group or non-iCTA group (neither since 11/2012 nor 10/2016). However, mean FU time was longer in the non-iCTA group (since 11/2012) compared to the iCTA group as it was introduced in 10/2016 (19.4 vs. 32.6 months, *p* < 0.001). Detailed patient characteristics are summarized in Table 1.

**Table 1:** Patient characteristics of all patients, patients without receiving iCTA (since 11/2012 and 10/2016) and patients receiving iCTA

Parameter	All patients (11/2012–12/2019) *n* = 270 (percentage)	No iCTA (11/2012–12/2019) *n* = 196 (percentage)	No iCTA (10/2016–12/2019) *n* = 53 (percentage)	iCTA (10/2016–12/2019) *n* = 74 (percentage)	^#^*p* value	^##^*p* value
Median age [years]	57.0	57.8	58.3	54.7	.719	.102
Age range [years]	18—81					
Gender (Female/Male)	197/73 (73.0/27.0)	138/58 (70.4/29.6)	42/11 (79.2/20.8)	59/15 (79.7/20.3)	.129	.559
Aneurysm location					.587	.280
MCA	157 (58.1)	116 (59.2)	35 (66.0)	41 (55.4)		
ICA	31 (11.5)	20 (10.2)	7 (13.2)	11 (14.9)		
ACoA	51 (18.9)	39 (19.9)	7 (13.2)	12 (16.2)		
PICA	3 (1.1)	3 (1.5)	1 (1.9)	0		
ACA	19 (7.0)	12 (6.1)	2 (3.8)	7 (9.5)		
PCoA	8 (3.0)	5 (2.6)	0	3 (4.1)		
SUCA	1 (0.4)	1 (0.5)	1 (1.9)	0		
Multiple aneurysms	85 (31.5)	60 (30.6)	18 (34.0)	25 (33.8)	.660	.983
Side (right/left)	93/127 (34.4/47.0)	65/93 (33.2/47.4)	28/18 (52.8/34.0)	34/28 (45.9/37.8)	.720	.850
Median size [mm]	6.7	6.8	5.0	6.6	.378	.215
Size range [mm]	2—20					
Temporary clipping	40 (14.8)	30 (15.3)	7 (13.2)	10 (13.5)	.848	.960
Mean FU time	28.9	32.6	18.1	19.3	.001	.779

### Intra- and interrater reliability

For the classification of aneurysm remnant according to the Sindou classification, substantial agreement was seen in rater 1’s intrarater assessment (Kendall τ-b = 0.950, 95% CI: 0.926 to 0.966, *p* < 0.001) and in rater 2’s assessment (Kendall τ-b = 0.972, 95% CI: 0.959 to 0.982, *p* < 0.001). The overall interrater reliability for the analysis of aneurysm remnant according to the Sindou classification was high (Kendall τ-b = 0.857; 95% CI: 0.792–0.902 to Kendall τ-b = 0.893; 95% CI: 0.844–0.927).

### Aneurysm remnants and clinical management

During surgery, ICGVA results led to clip repositioning in 12/270 patients – 4/74 (5.4%) in the iCTA group and 8/196 (4.1%) in the non-iCTA group.

**Fig. 2:**
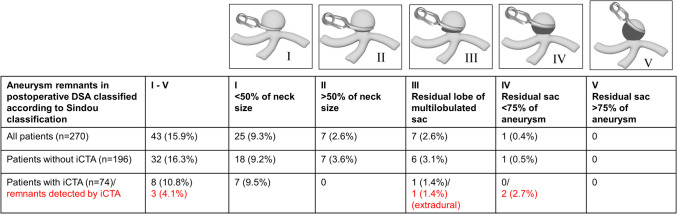
Aneurysm remnants classified according the Sindou classification

In the non-iCTA group, postoperative DSA detected in the majority of cases small remnants SG I-II (SG I: *n* = 18, 9.2%; SG II: *n* = 7, 3.6.%). Larger aneurysm remnants were found in 7 patients (SG III: *n* = 6, 3.1%, SG IV: *n* = 1, 0.5%). Illustration of the remnants is shown in Fig. 2.

In the iCTA group, the majority of patients similarly presented small SG I aneurysm remnants (*n* = 7, 9.5%). A larger remnant was only found in one patient (SG III: *n* = 1, 1.4%) exhibiting an ICA aneurysm at the branch of the ophthalmic artery which was not detected by iCTA due to limited visibility at the clinoid process. Despite regular ICGVA results, iCTA was able to detect SG III and IV remnants in 4.1% of patients intraoperatively—two SG IV remnants (2.7%) and one SG III remnant (1.4%) correctly (Fig. 2).

### Illustrative cases

**Fig. 3:**
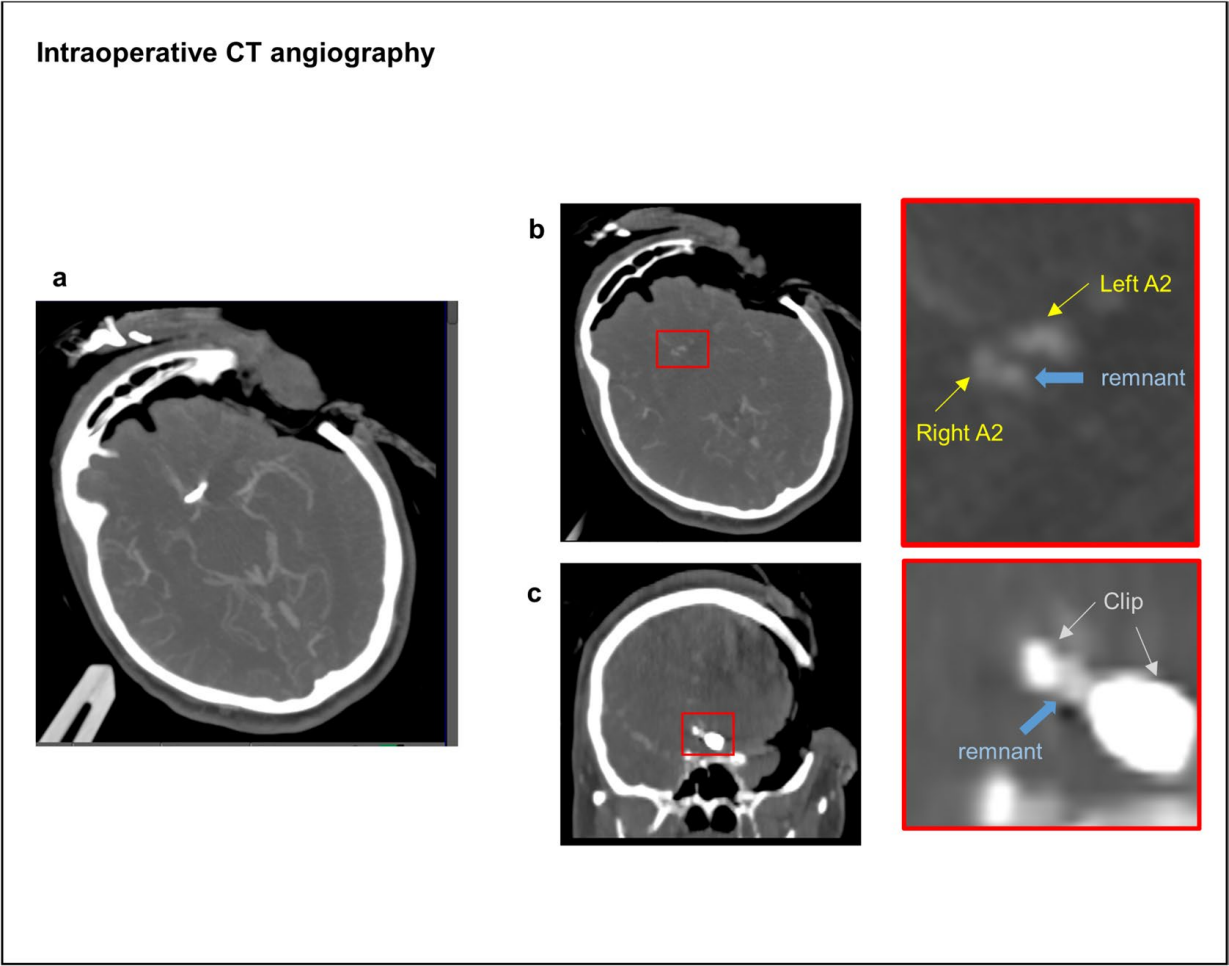
A 51-year-old female patient with a 6 mm polylobulated Acom aneurysm was clipped from the left side. ICG-videoangiography showed a sufficient obliteration of the aneurysm and the A2 branches were contrasted regularly. However, iCTA showed an aneurysm remnant at the tip of the clip resulting in a Sindou grade IV. Aneurysm remnant is shown in axial (**a**, **b**) and coronar (**c**) plane

In detail, one 51-year-old female patient with a 6 mm polylobulated Acom aneurysm was clipped from the left side with intraoperative rupture (Fig. 3). ICGVA showed a sufficient obliteration of the aneurysm. However, iCTA revealed a remnant at the tip of the clip. Due to limited view, it was not possible to reposition the clip with preserving the contralateral A2 branch. Therefore, an angiography was performed postoperatively, which confirmed a SG IV remnant. A second successful surgery from the contralateral side was performed resulting in a complete aneurysm occlusion. Another 53-year-old male patient with a 10 mm Acom aneurysm with calcification in the parent vessel also revealed despite normal ICGVA results a remnant in iCTA after clipping. As intraoperative clip repositioning was not possible due to insufficient visualization of the aneurysm neck, the patient was successfully clipped from the contralateral side on the next day exhibiting no remnant in second postoperative angiography. In a 47-year-old female patient with a Pcom aneurysm, iCTA showed a remnant in the caudal part of the aneurysm. Postoperative angiography revealed that the SG III remnant was located in the extradural space (Supplementary Fig. 1). The patient was recommended endovascular therapy. However, she refused and FU CT-angiography after 42.9 months did not reveal a remnant growth.

Additionally, a 52-year-old female patient exhibited a fusiform, twisted aneurysm at the ICA siphon, which was not feasible to clip without occlusion of ICA. This was tolerated due to excellent collateralization confirmed by iCTA and unremarkable intraoperative neuromonitoring (IONM). Also, a 60-year-old male patient suffered from a large calcified and partially thrombosed MCA aneurysm. After first clip positioning, iCTA showed that the M2-branches were not contrasted. Therefore, the aneurysm sack had to be excised due to torquing of the parent vessel. A second iCTA showed regular contrast enhancement of the M2 branches and a normalized intraoperative CT perfusion.

### Surgery- and outcome-related parameters

Surgery- and outcome-related parameters are shown in Table 2. In the iCTA group the frequency of aneurysm remnants SG I-V (*n* = 8) was slightly lower than in the non-iCTA group (*n* = 32) (10.8 vs. 16.3%, *p* < 0.173). The frequency of SG III-V remnants was lower in the iCTA group (since 11/2012) (*n* = 1) than in the non-iCTA group (since 11/2012) (*n* = 7) (1.4 vs. 3.6%, *p* < 0.306) with a mean remnant size of 1.1 and 1.2 mm. Also, a stenosis of the parent vessel was detected less frequently in the iCTA group (*n* = 1) compared to the non-iCTA group (since 11/2012) (*n* = 14) (1.4 vs. 7.1%, *p* < 0.052). The rate of permanent deficit was comparable in both groups (iCTA, *n* = 3 vs. non-iCTA since 11/2012, *n* = 9; 4.1 vs. 4.6%, *p* < 0.353).

**Table 2:** Surgery- and Outcome-related parameters of all patients, patients without receiving iCTA (since 11/2012 and 10/2016) and patients receiving iCTA

Parameter	All patients (11/2012–12/2019) *n* = 270 (percentage)	No iCTA (11/2012–12/2019) *n* = 196 (percentage)	No iCTA (10/2016–12/2019) *n* = 53 (percentage)	iCTA (10/2016–12/2019) *n* = 74 (percentage)	^#^*p* value	^##^*p* value
Remnant Sindou I-V	40 (14.8)	32 (16.3)	7 (13.2)	8 (10.8)	.173	.805
Remnant Sindou III-V	8 (3.3)	7 (3.6)	0	1 (1.4)	.306	NA
Mean remnant size [mm]	1.2	1.2	0.4	1.1	.471	.001
Stenosis in postop. DSA	15 (5.6)	14 (7.1)	1 (1.9)	1 (1.4)	.043	.662
Permanent deficit	11 (4.0)	9 (4.6)	1 (0.8)	3 (4.1)	.353	.303

In the non-iCTA subgroup compared to the iCTA group since 10/2016, both the rate of SG I-V remnants (iCTA *n* = 8 vs. non-iCTA *n* = 7, 10.8 vs. 13.2, *p* < 0.805) and the rate of stenosis in postoperative DSA (iCTA *n* = 1 vs. non-iCTA *n* = 1, 1.4 vs. 1.9, *p* < 0.662) were similar. The rate of permanent deficit was slightly increased in the iCTA group, but not statistically significant (iCTA *n* = 3 vs. non-iCTA *n* = 1, 4.1 vs. 0.8, *p* < 0.303). There was one SG IV remnant in the iCTA group compared to none in the non-iCTA group since 10/2016. Therefore, the mean remnant size differed significantly between the two groups (iCTA: 1.1 mm vs. non-iCTA: 0.4 mm, *p* < 0.001).

A table summarizing the specific characteristics of patients with missed remnants by iCTA is provided in supplementary Table 1.

The estimated number needed to treat to detect one SG III-V remnant through application of ICGVA and additionally iCTA was statistically determined to be 25 cases and the absolute risk reduction using iCTA was 4%

### Independent predictors for aneurysm remnant

Relevant patient- or surgery-related variables were evaluated stepwise in bivariate and multivariate analyses (Table 3). Accordingly, location MCA vs. ACoA (*p* < 0.026) and MCA vs. PCoA (*p* < 0.005) were independent risk factors for Sindou I-V remnants in DSA; MCA vs. PCoA remained statistically significant in the multivariate analysis (*p* < 0.002). Risk factor for Sindou III-V remnants in DSA was location MCA vs. ACoA (*p* < 0.046).

**Table 3:** Independent predictors for aneurysm remnants Sindou I-V and Sindou III-V in postoperative DSA

	Sindou I-V remnant in DSA		Sindou III-V remnant in DSA	
	*p* value	OR/95%CI	*p* value	OR/95%CI
Univariate analysis				
age (continuously)	NS (*p* =.47)		NS (*p* =.49)	
gender (male vs. female)	NS (*p* =.49)		NS (*p* =.90)	
side (right vs. left)	NS (*p* =.10)		NS (*p* =.46)	
surgeon	NS (*p* =.98)		NS (*p* =.92)	
aneurysm size (continuously)	NS (*p* =.78)		NS (*p* =.38)	
(< 5 mm vs. ≥ 5 mm)	NS (*p* =.41)		NS (*p* =.66)	
aneurysm location (overall)	NS (*p* =.06)		NS (*p* =.74)	
MCA vs. ICA	NS (*p* =.19)		NS (*p* =.22)	
MCA vs. ACoA	*p* <.026	2.534/1.116 to 5.752	*p* <.046	2.735/1.016 to 7.359
MCA vs. PICA	NS (*p* =.26)		NS (*p* =.99)	
MCA vs. ACA	NS (*p* =.63)		NS (*p* =.85)	
MCA vs. PCoA	*p* <.005	8.235/1.885 to 35.979	NS (*p* =.51)	
MCA vs. SUCA	NS (*p* =.99)		NS (*p* =.99)	
Multiple aneurysms	NS (*p* =.58)		NS (*p* =.71)	
Temporary clipping (yes/no)	NS (*p* =.97)		NS (*p* =.85)	
iCTA	NS (*p* =.26)		NS (*p* =.36)	
Multivariate analysis				
side (right vs. left)	NS (*p* =.07)		NS (*p* =.16)	
aneurysm size (< 5 mm vs. ≥ 5 mm)	NS (*p* =.23)		NS (*p* =.74)	
aneurysm location	NS (*p* =.06)		NS (*p* =.74)	
MCA vs. ICA	NS (*p* =.19)		NS (*p* =.17)	
MCA vs. ACoA	NS (*p* =.70)		NS (*p* =.37)	
MCA vs. PICA	NS (*p* =.26)		NS (*p* =.99)	
MCA vs. ACA	NS (*p* =.11)		NS (*p* =.43)	
MCA vs. PCoA	*p* <.002	12.397/2.556 to 60.139	NS (*p* =.40)	
MCA vs. SUCA	NS (*p* =.99)		NS (*p* =.99)	
Temporary clipping (yes/no)	NS (*p* =.49)		NS (*p* =.42)	
iCTA	NS (*p* =.51)		NS (*p* =.42)	

### Sensitivity and Specificity

The specificity, sensitivity, and accuracy for detection of a SG I-V remnant by iCTA were 100%, 15.8%, and 74.8% and for detection of a SG III-V remnant by iCTA were 100%, 75%, and 98.6%, respectively (Table 4).

**Table 4:** Sensitivity, specificity, PPV, NPV, FN, FP and accuracy for the detection of a Sindou grade I-V and Sindou grade III-V remnant by iCTA

Parameter	Sens [%]	Spec [%]	PPV [%]	NPV [%]	FN [%]	FP [%]	Accuracy [%]
iCTA (Sindou I-V remnants)	15.8	100	100	77.5	84.2	0	78.4
iCTA (Sindou III-V remnants)	75.0	100	100	98.6	25.0	0	98.6

iCTA, intraoperative CT angiography; Sens, sensitivity; Spec, specificity; PPV, positive predictive value; NPV, negative predictive value; FN, false negative; FP, false positive

### Radiation exposure

The mean ED was 1.46 mSv for iCTA and 1.50 mSv for DSA.

### Therapeutic intervention and follow-up of aneurysm remnants

**Fig. 4:**
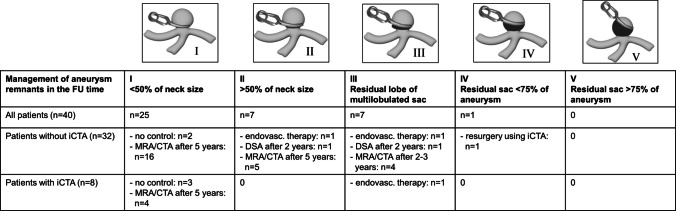
Clinical management of the aneurysm remnants

In the interdisciplinary neurovascular board, postoperative angiography of all patients was reviewed and indication for resurgery, endovascular therapy or FU imaging was determined (Fig. 4). The patient with a SG IV aneurysm remnant (non-iCTA group) was successfully operated using iCTA. One patient with SG II and two patients with SG III aneurysm remnants were treated by endovascular therapy. All other patients received FU imaging by MRA or CTA. After a mean FU time of 28.9 months, neither growth of the aneurysm remnant nor subarachnoid hemorrhage of the aneurysm remnant could be detected in the remaining 36 patients.

## Discussion

In general, aneurysm remnants after clipping of unruptured intracranial aneurysms are very rare [1, 21, 22, 25] and in the majority of cases, small remnants may also be attributed to branching perforators or calcifications on the parent vessel in order to avoid intraoperative vessel occlusion. Therefore, it remains indeterminate if SG I or II remnants might have an impact on clinical relevance in the FU time.

In this study, we only included patients who underwent postoperative angiography in order to enable a transparent analysis of the detected aneurysm remnants. Also, we excluded patients with ruptured aneurysms to prevent potential confounders in terms of blood in the subarachnoid space in order to achieve optimal conditions for interpretation of intraoperative CTA results.

In our cohort of 270 patients undergoing aneurysm clipping, 15.9% of the patients exhibited aneurysm remnants in the immediate postoperative DSA which is in line with other studies depending on the postoperative imaging modality of visualization [1, 21, 22, 25]. However, the majority of remnants were small (SG I and II). By introduction of numerous technical improvements in the last decade like ICGVA, microdoppler or intraoperative DSA, an improved intraoperative visualization of vascular anatomy could be achieved [2, 9, 12, 34, 35]. ICGVA has the advantage of simple feasibility and low costs of the technique. A benefit rate of nearly 15% of the procedures was described and argues for routine application during aneurysm clipping. In this study, we observed intraoperative clip reposition in 4.5% of all cases according to ICGVA results. However, former studies demonstrated that in up to 10% of patients, small aneurysm remnants can be overlooked intraoperatively by ICGVA [36]. In a recent study, it could be shown that intraoperative DSA revealed aneurysm remnants in 7/120 (5.8%) which were overlooked by ICGVA, especially in the anterior communicating artery complex [31]. Thus, the combination of ICGVA and intraoperative angiography may prove most effective for maximizing the efficacy of aneurysm surgery [49].

A cost–benefit analysis of iCTA, DSA and ICGVA for detecting cerebral aneurysm remnants involves assessing their effectiveness, costs and risks. Regarding diagnostic accuracy, DSA remains the gold-standard according to the highest spatial resolution compared to iCTA which is less sensitive for very small remnants or near clip artifacts. ICGVA provides real-time vascular assessment, however it is limited to the surgical field. On the subject of cost considerations, DSA exhibits the highest costs due to angiography suite use, contrast agents, radiation exposure and hospital resources compared to iCTA which requires lower resource and personnel requirements. ICGVA costs mainly arises from ICG dye and microscope integration. Concerning safety and risks iCTA is non-invasive, but exposes patients to radiation and contrast, however it is less invasive compared to DSA and lacks periinterventional risks e.g. stroke or hematoma. Further, it allows full cerebral vasculature assessment compared to ICGVA.

Radiation exposure of iCTA was comparable to DSA. Even though the iCTA protocol has been optimized [16], we believe that further dose reduction of iCTA can be achieved when considering the installed iCT scanner generation in this study. In detail, our iCT is a 128-slice CT scanner which is only equipped with one x-ray tube (single-source CT). It is well known, that dual-source CT scanners (two x-ray tubes) yield significantly lower radiation dose while maintaining good image quality with median ED values ranging between 0.3–0.5 mSv for routine carotid CTA (head and neck) according to recent data of Forbrig and colleagues [18]. Also, in their study, the radiation dose of carotid CTA carried out on a similar single-source CT was substantially lower (median 0.75 mSv) compared to our data. To note, dosimetric comparison of iCTA and routine CTA is limited, as the iCTA protocol differs substantially to allow for sufficient image quality in this special intraoperative CT setting (e.g., artifacts due to air and fluids as well as metallic implants and instruments, respectively).

Up to now, studies for the intraoperative detection of aneurysm remnants by iCTA were missing. Our previous reports have investigated iCT feasibility and applicability into routine neurovascular procedures [14, 37–39]. In a recent study, we could show in a large cohort that there was a stepwise improvement in the rate of complete aneurysm occlusion, postoperative ischemia and neurological outcome in the last 20 years [40]. The current study could show that iCTA was able to detect SG III and IV remnants correctly in 3 of 4 patients resulting in a sensitivity, specificity, and accuracy of 75%, 100%, and 98.6%, respectively. This is in line with other studies showing a pooled sensitivity of 71% and specificity of 94% for identifying residual or recurrent aneurysms by postoperative CTA in comparison to DSA [42]. For aneurysm remnants < 2 mm, the sensitivity has been shown to be even lower [6–8, 13, 45] which is in line with the low sensitivity of 15.8%, and accuracy of 74.8% for SG I-V remnants in the present study. Novel methods like dynamic and bone subtraction CTA can improve on the sensitivity of CTA for aneurysms or aneurysm remnants, but warrant further investigation [48]. Although, iCTA was able to detect remnants intraoperatively, immediate clip repositioning was not possible due to anatomical reasons in this study. However, we suppose that iCTA results could have an impact on the surgical strategy.

For data consistency, we analyzed a subgroup of patients in the non-iCTA group since 10/2016 in comparison to the iCTA group. In this subgroup of 53 patients, the aneurysm size was slightly smaller in the iCTA group (5 vs. 6.6 mm) and the location was more frequently in the anterior complex and posterior communicating artery (29.8% vs. 17%) implicating that more complex aneurysms might be clipped using iCTA. This was confirmed by the uni- and multivariate analysis, which showed that aneurysm located in the ACoA and PCoA were significantly more likely to exhibit Sindou I-V and Sindou III-V remnants. However, this warrants a prospective study to address on the question.

In our previous study, a statistically significant improved rate of radiologically confirmed complete aneurysm occlusion could be achieved in the last two decades, which pertained the first cohort (12/2000–12/2008) vs. the third cohort (01/2016–12/2019) [40]. In the present study, the univariate analysis could not reveal any risk factors neither for SG I-V nor SG III/IV remnants which may be attributed to the limited patient number. Patient-/aneurysm-related factors significantly associated with aneurysm remnants were reported to be atherosclerosis [28], aneurysm size [2, 23, 41] or location [2, 23, 41]. Also, large to giant aneurysms with complex morphology were also shown to be prone to aneurysm remnants [27]. Tsutsumi et al. identified the presence of multiple aneurysms at initial presentation and residual aneurysm after clip ligation as important risk factors for recurrence [46]. Also, age less than 45 years was shown to be an independent risk factor for remnant growth in the study of Jabbarli et al. [22, 23].

In several studies no association between remnant size and remnant growth was identified [5, 15, 30]. However, in a multicenter analysis of rupture risk after clipping, the estimates of degree of aneurysm occlusion were highly predictive for rerupture [24]. It seems plausible that rupture risk is more likely due to a residual pathological vessel wall than a completely reconstituted healthy vessel wall by clipping [32]. Further, histopathological analysis of human clipped aneurysms revealed that complete re-endothelialization and neointima formation occurred only in aneurysms with complete elimination of the pathological vessel wall [26]. Therefore, we also accounted SG III aneurysms as accessible for retreatment given the fact of a remaining aneurysm lobe which was also described in the original publication of Sindou et al. [41].

After mean FU of 29 months neither the SG I-II remnants nor the remaining five SG III-V remnants did reveal growth or rupture in our study. A late angiographic FU review showed that among 135 (91.8%) clipped aneurysms without initial residua, 2 (1.5%) aneurysms recurred at a mean of 4.4 ± 1.6 years postsurgery (range 2.6–9.7 years) and 12 (8.2%) clipped aneurysms showed remnants which demonstrated growth in 2 cases (16.7%) at FU visits [11]. Therefore, a long-term evaluation of the remnants in our study is warranted.

In summary, we consider that relevant aneurysm remnants can be sufficiently identified by intraoperative application by ICGVA in the majority of cases. For smaller remnants, e. g. SG I – II, iCTA is not advantageous compared to iDSA according to the low sensitivity. However, iCTA can be beneficial to detect Sindou III to V remnants in complex aneurysms where visualization of the neck is limited or calcification or partial thrombosis, especially when iDSA is not available.

### Limitations

Limitations of our study are the retrospective and single center design as well as the rather short FU period to detect aneurysm growth in the interval – however the recommended time interval for control of SG I – II aneurysms was determined between two to five years.

Further, a potential selection bias could be due to the exclusion of patients who did not undergo postoperative DSA according to contraindications for DSA, missing consent and lost to FU. The exclusion may lead to an overrepresentation of patients with more favourable outcomes or those whose clinical condition allowed for postoperative imaging. This bias could limit the generalizability of our findings and may underestimate the true incidence of aneurysm remnants.

Compared to ICGVA, iCTA is unfavourable regarding radiation exposure. However, iCTA compromises less radiation doses than intraoperative angiography and efforts were made in the present study to further optimize the iCTA protocol (e.g. reduction of tube output and voltage) resulting in a significant decrease of the effective dose from 2.97 mSv to 1.46 mSv [16].

## Conclusion

Aneurysm remnants after clipping are rare. As detected by DSA, in the majority of cases the remnants are small (SGI-II) and do not seem to harbour a risk of growth/rupture during short-term follow-up. For detection of a SG III-V remnant using iCTA, the specificity, sensitivity, and accuracy were 100%, 75%, and 98.6%, respectively. Therefore, we consider that in patients with complex configurated calcified, or partially thrombosed aneurysms iCTA could be beneficial besides ICGVA to intraoperatively identify SG III-V remnants and to adjust the surgical strategy.

## Supplementary Information

Below is the link to the electronic supplementary material.

Supplementary file1 (PDF 129 KB)

Supplementary file2 (DOCX 14 KB)

## Data Availability

The data that support the findings of this study are available from the corresponding author, Jun Thorsteinsdottir, upon reasonable request. No datasets were generated or analysed during the current study.

## Abbreviations

ACA: Anterior cerebral artery
ACoA: Anterior communicating artery
CBP: Cerebral blood perfusion
CBV: Cerebral blood volume
CSF: Cerebrospinal fluid
CT: Computed tomography
DSA: Digital subtraction angiography
ICA: Internal carotid artery
ICGVA: Indocyanine green videoangiography
iCT: Intraoperative CT
iCTA: Intraoperative CT angiography
iCTP: Intraoperative CT perfusion
FU: Follow-up
GOS: Glasgow outcome scale
INVB: Interdisciplinary neurovascular board
IONM: Intraoperative neuromonitoring
MCA: Middle cerebral artery
MEP: Motor evoked potential
MRI: Magnetic resonance imaging
mRS: Modified Rankin score
MTT: Mean transit time
PCoA: Posterior communicating artery
PICA: Posterior inferior cerebellar artery
SEP: Somatosensory evoked potential
SG: Sindou Grade
SUCA: Superior cerebellar artery
