# Supplementary material for: Detection of remnants in clipped unruptured intracranial aneurysms by intraoperative CT-angiography and postoperative DSA: clinical relevance and follow-up
Source: Acta Neurochir (Wien). 2025 Apr 17;167(1):109. doi: 10.1007/s00701-025-06518-3 (PMC12003565; doi:10.1007/s00701-025-06518-3)
Supplement: Supplementary file 2 — Supplementary file2 (DOCX 14 KB) [file 701_2025_6518_MOESM2_ESM.docx]

| **Parameter** | **Patients with remnants missed in iCTA** |
| --- | --- |
| Median age [years] | 49.0 |
| Age range [years] | 32 – 67 |
| Gender (Female/Male) | 7/1 (87.5/12.5) |
| Aneurysm location |  |
| MCA | 1 (12.5) |
| ICA | 2 (25.0) |
| ACoA | 1 (12.5) |
| PICA | 0 |
| ACA | 3 (37.5) |
| PCoA | 1 (12.5) |
| SUCA | 0 |
| Side (right/ left) | 2/5 (62.5/25.0) |
| Median size [mm] | 5.5 |
| Size range [mm] | 4 – 8 |
| Mean remnant size [mm] | 1.9 |
| Temporary clipping | 0 |
| Mean FU time [months] | 8.5 |

**Supplementary Table 1:** Patient characteristics of patients with aneurysm remnants which were missed in iCTA.
